# P09 Non-allergist Removal of Incorrect Penicillin Allergy Labels (RIPAL) in a UK hospital

**DOI:** 10.1093/jacamr/dlad066.013

**Published:** 2023-06-14

**Authors:** Daniel Hearsey, Shuayb Elkhalifa, Jonathan Sandoe, Michael Wilcock, Rhys Owens, Bethan Gay, Charlotte Wildblood, Jane Mendonca, Nicola Leigh, Neil Powell

**Affiliations:** Royal Cornwall Hospital Trust, Truro, Cornwall, UK; Allergy and Immunology Department, Respiratory Institute, Cleveland Clinic Abu Dhabi, United Arab Emirates; Leeds Institute of Medical Research, University of Leeds, Leeds, UK; Royal Cornwall Hospital Trust, Truro, Cornwall, UK; Royal Cornwall Hospital Trust, Truro, Cornwall, UK; Royal Cornwall Hospital Trust, Truro, Cornwall, UK; Royal Cornwall Hospital Trust, Truro, Cornwall, UK; Royal Cornwall Hospital Trust, Truro, Cornwall, UK; Royal Cornwall Hospital Trust, Truro, Cornwall, UK; Royal Cornwall Hospital Trust, Truro, Cornwall, UK

## Abstract

**Objectives:**

We piloted a pharmacist-led multidisciplinary penicillin allergy de-labelling (PADL) daily ward round to determine the opportunity for PADL in a UK hospital.

**Methods:**

A daily ward round, delivered by either an antibiotic pharmacist or a junior doctor, identified adult medical and surgical patients between 7 November 2022 and 31 January 2023 with a penicillin allergy (penA) record that was preventing first-line penicillin use. An allergy history was taken before risk stratifying likelihood of future harm from penicillin re-exposure and an allergy testing method was determined [de-label on history (DOH) or after direct drug provocation testing (DDPT)]. Following successful de-label, in agreement with the responsible clinician, the antibiotic was switched to a penicillin antibiotic.

**Results:**

Of 7214 inpatients during the study period, 1133 (15.7%) had a penA record. Of 285 allergy histories taken, 105 (36.8%) met high-risk criteria, 45 (15.8%) met low-risk criteria eligible for DOH, 73 (25.6%) met low-risk criteria eligible for DDPT and we were unable to obtain a history from 62 (21.8%) patients. Of 45 low-risk patients eligible for DOH, 40 (88.9%) were de-labelled of which 24 (53.3%) were switched to a penicillin antibiotic. Of 73 patients with a low-risk allergy history eligible for DOC, 16 (21.9%) received DDPT, of which nine were switched to a penicillin antibiotic to complete their antibiotic course. Two DOH patients experienced harm (thrush within the first 5 days and delayed skin reaction after day 5), none of the DPTs had a reaction by day 5. The switches resulted in 170 DDDs of penicillin use (predominantly amoxicillin) and reduced alternative antibiotic use by 174 DDDs (predominantly meropenem and levofloxacin).

**Conclusions:**

Penicillin allergy de-labelling patient pathway delivered by pharmacists and junior doctors was safe and effective and well accepted by patients and the wider clinical teams. Further work is required to optimize and streamline these patient pathways to maximize patient and health system benefit.

